# Which treatment for upper respiratory tract infections?

**DOI:** 10.1186/1824-7288-41-S2-A31

**Published:** 2015-09-30

**Authors:** Pietro Ferrara, Costanza Cutrona, Annamaria Sbordone

**Affiliations:** 1 Institute of Pediatrics, Catholic University of Sacred Heart, Rome, Italy; 2 Campus Bio-Medico University, Rome, Italy

## 

Upper Respiratory Tract Infections (URTIs) include rhinosinusitis, acute otitis media (AOM), pharyngotonsillitis and laryngitis [1]. Viruses are responsible for the great majority of URTIs therefore antimicrobial treatment is not always required [2]. Paracetamol (7-15 mg/kg/dose) and ibuprofen (4-10 mg/kg/dose) are considered as the standard analgesics [3].

Regarding rhinosinusitis, clinicians should suspect a bacterial etiology when a child presents with persistent, worsening or severe illness. *S. pneumoniae, H. influenzae* and *M. catarrhalis* are the most common isolated bacteria. Amoxicillin (50 mg/kg/day) alone or with clavulanate is the first line antibiotic. Ceftriaxone (50 mg/kg/day) should be given to children who cannot take oral medications. Duration of the treatment varies from 10 to 28 days. The antibiotic may be changed if the symptoms get worse or do not improve within 72 hours (cefixime 8 mg/kg/day) [4-6].

AOM is an inflammatory disease of the middle ear involving the tympanic cavity frequently caused by *S. pneumoniae, H. influenzae* and *M. catarrhalis* . Diagnostic criteria for AOM are:

• moderate to severe bulging of TM or new onset of othorrea not due to acute otitis externa;

• mild bulging of the TM and recent onset of earache or intense erythema of the TM.

Pediatricians should prescribe antibiotics in children aged <6 months with both severe and moderate presentations, in children aged between 6 and 24 months with severe presentation in both unilateral and bilateral AOM or in those with moderate presentation in bilateral AOM. Children aged > 24 months need antibiotic therapy only when the presentation is severe [7,8]. Amoxicillin (50 mg/kg/day) alone or with clavulanate is the first choice in moderate and severe presentation respectively. Alternative initial antibiotics include cefaclor (40-50 mg/kg/day) and cefuroxime-axetil (30 mg/kg/day) or cefpodoxima-proxetil (8 mg/kg/day) respectively. Ceftriaxone (50 mg/kg/day) can be given to children who cannot take oral medications or when the symptoms do not improve within 72 hours [9]. The duration of the treatment may vary from 5 to 10 days [8].

Most pharyngitis episodes are caused by viruses. Antibiotic therapy is recommended in every child with microbiologically documented *group A β-hemolytic streptococcus* pharyngitis (37%). The first-line treatment is amoxicillin (50 mg/kg/day for 10 days). In non-compliant cases, cefaclor (40 mg/kg/day) or cefuroxime-axetil (20-30 mg/kg/day) may be administrated [10].

Epiglottitis is a supraglottic laryngitis. It may be caused by *S. pneumonia, S. aureus, β-hemolytic streptococcus* and *H. influenzae*. The priority is airway management followed by antibiotic (ceftriaxone 50-75 mg/kg/day) treatment and steroids [11].
